# Pseudoaneurysm of the Common Carotid Artery in an Infant due to Swallowed Fish Bone

**DOI:** 10.1155/2015/903150

**Published:** 2015-12-09

**Authors:** Moulion Tapouh Jean Roger, Fokou Marcus, Fongang Emmanuel, Moifo Boniface, Juimo Alain Georges

**Affiliations:** ^1^Yaounde University Hospital Center, Yaounde, Cameroon; ^2^Department of Surgery, Yaounde General Hospital, Yaounde, Cameroon; ^3^Radiology Department, Yaounde General Hospital, Yaounde, Cameroon; ^4^Faculty of Medicine and Biomedical Sciences (FMBS), Yaounde, Cameroon; ^5^Radiology Department, Yaounde Gynaeco-Obstetric and Paediatric Hospital, Yaounde, Cameroon

## Abstract

Carotid artery pseudoaneurysm is a rare condition, particularly in the paediatric population. Only about 30 cases of carotid artery aneurysms in infants have been published until now. This paper reports the case of a giant pseudoaneurysm of the left common carotid artery due to swallowed fish bone by an 8-year-old boy. This pseudoaneurysm was 5.5 cm transverse-diameter and resulted in severe respiratory distress. It was treated by resection and end-to-end anastomosis with satisfactory outcome after one-year follow-up. To the best of our knowledge, this is the largest carotid artery pseudoaneurysm ever described in children.

## 1. Introduction

Cervical masses in infants are frequent during routine clinical practice. They are usually congenital or of infectious origin and are often due to benign adenitis [1–3]. Vascular abnormalities, such as extracranial carotid aneurysms, are very uncommon. These aneurysms could be true aneurysms involving all the layers of the arterial wall or pseudoaneurysms in which there is no organic wall [4–6]. To our knowledge just about 30 cases of carotid artery aneurysms in infants have been published, a third of which were posttraumatic pseudoaneurysms which were either due to an accident or iatrogenic [5, 7, 8]. We report the case of an 8-year-old boy with no particular past history who underwent imaging for a suspected giant pseudoaneurysm of the left common carotid artery. This case is peculiar because of the exceptional aetiology which was the ingestion of an unnoticed fish bone.

## 2. Observation

**Figure 1:**
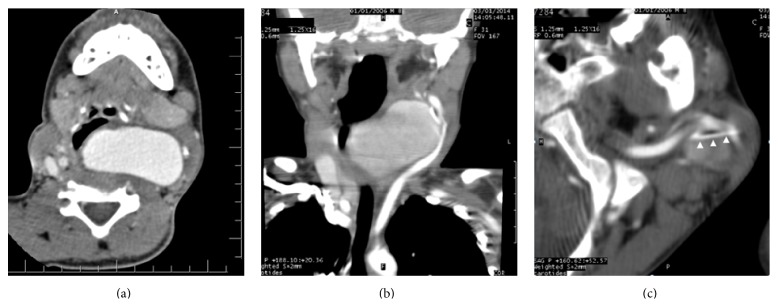
Axial (a) and coronal reformatted (b) contrast-enhanced CT scan images of the neck showing a saccular dilatation of the left common carotid artery with mass effect on the trachea and adjacent vascular structures. Axial bone window image (c) along the upper portion of the aneurysm shows a linear hyperdensity denoted with arrow-heads (later confirmed to be a fish bone).

An 8-year-old boy was investigated in the radiology department for a left-sided neck swelling which was observed 3 weeks earlier and was gradually increased in size. Physical examination revealed a painless pulsatile left-lateral cervical mass with a smooth outline, mild edema of the left hemiface, and a normal temperature. The infant looked generally well. A systolic murmur was present and suggestive of aneurysm. A contrast-enhanced computed tomography (CT) scan of the neck was performed (Figure 1) and showed a 42 mm × 55 mm × 28 mm (*H* × *W* × AP) saccular dilatation of the left common carotid artery situated 20 mm proximally to the bifurcation. There was mass effect on adjacent structures notably the oesophagus and the trachea without significant stenosis. On reformatted images, a 45 mm long linear hyperdensity was depicted inside the aneurysm.

**Figure 2:**
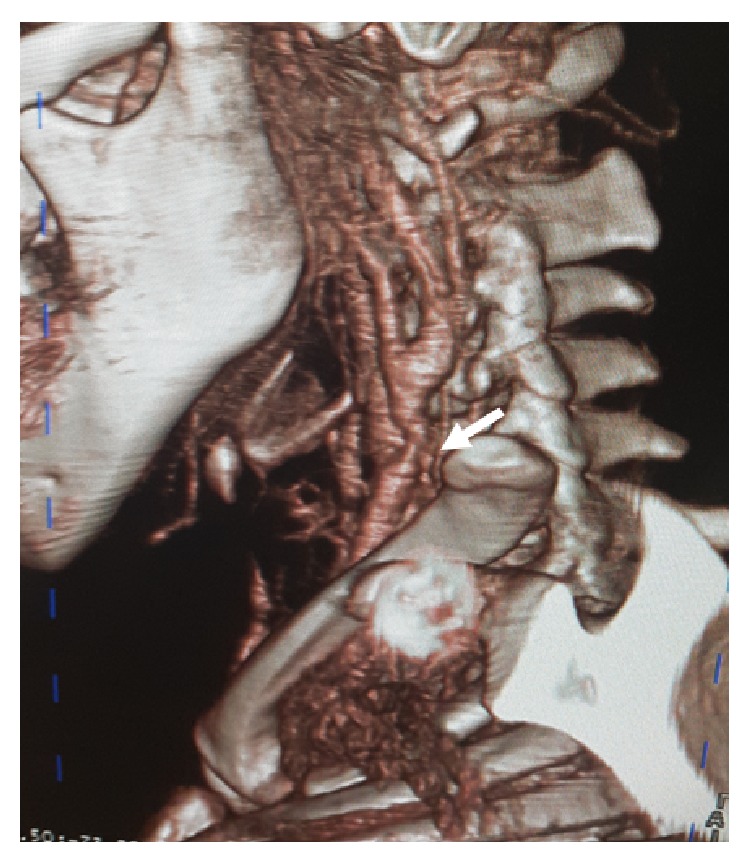
VRT CT picture at one-year follow-up. The common carotid is permeable without any residual dilation, with some mild scar narrowing at the anastomotic site (white arrow).

The infant was admitted in the surgery department for a comprehensive preoperative work-up. A severe inspiratory dyspnea ensued which led to patient transfer to the intensive care unit where endotracheal intubation was performed. Heparin was administered and an emergency surgical operation was performed through a left posterior cleidomastoid incision, followed by clamping of the ipsilateral common, internal, and external carotid arteries. The aneurysmal sac was resected and a fish bone was found inside which had perforated the common carotid wall. An end-to-end anastomosis was carried out using Prolene 5/0 and cerebral blood-flow was reestablished. On day 2 after surgery there was complete regression of the dyspnea and one year later the patient was completely asymptomatic (Figure 2).

## 3. Discussion

Pseudoaneurysm is a hematoma that forms as the result of a leaking hole in an artery. It arises when a tear occurs in the arterial wall allowing blood to leak into the surrounding tissue, with persistent communication between the arterial lumen and the blood-filled cavity [9]. The main difference between true aneurysm and pseudoaneurysm is the lack of adventitia in pseudoaneurysm, where the whole wall is lacerated [8].

Pseudoaneurysms are particularly uncommon in the paediatric population. In a study of 57 659 infants with blunt trauma, Lew et al. reported only 15 cases (0.03%) of blunt carotid trauma [10] which are known to be potentially causative of pseudoaneurysms [11].

They generally are caused by direct arterial injury, which may result from trauma, vascular diagnostic or interventional procedures, adenotonsillectomy, and, in a lesser extent, deep infections [12]. Posttraumatic pseudoaneurysms of the carotid arteries are secondary to penetrating wounds or neck contusions. In these cases the tunica intima or media or the entire arterial wall is affected, resulting in extravasation into surrounding tissues [4, 7]. Iatrogenic pseudoaneurysms occur when the arterial wall is accidentally injured by a suture running too deeply [13] or a needle inserted for lymph node aspiration, for instance [9].

In our case, the child swallowed a fish bone two months before the cervical mass appeared. The fish bone perforated the wall of the oropharynx and injured the common carotid artery resulting in extravasation of blood into adjacent tissues. To our knowledge, this is the first report of a common carotid artery pseudoaneurysm resulting from food intake accident.

We found only 13 cases of noninfectious carotid pseudoaneurysms reported in the paediatric population from which 8 were iatrogenic (Table 1).

The most common site of extracranial carotid artery pseudoaneurysms is the external carotid artery [5, 14] while aneurysms often involve the bifurcation of the common carotid artery. In this case, the pseudoaneurysm was located 20 mm proximal to the carotid bifurcation.

Extracranial carotid arteries aneurysms (either true or false) can be asymptomatic or present as a pulsating cervical mass [15]. This mass can compress the upper airways leading to dyspnoea [16] as in this case. Rarely, it can lead to a recurrent laryngeal nerve palsy [14], dysphagia, Horner syndrome, neck pain, or thromboembolic conditions (cerebral ischemia). Less frequent presentations include a mass mimicking a peritonsillar abscess, epistaxis, nasopharyngeal mass, paranasal sinus mass, and a pharyngeal mass [17]. The onset of neck pain should prompt the clinician to search for complications such as rupture, mural thrombosis, or dissection [5, 7].

The diagnosis of pseudoaneurysms may be made with Doppler ultrasonography, CT Angiography (CTA), magnetic resonance imaging (MRI), and angiography [5]. Conventional radiographs may show nonspecific findings like a homogeneous cervical opacity of water density with smooth outline that may be calcified. Doppler ultrasonography would confirm this to be of vascular origin and permit blood-flow assessment but it does not give sufficient information to plan management decision [18]. On CTA, pseudoaneurysm is a mass that is enhanced homogeneously in continuity with a blood vessel. This study will permit an assessment of surrounding structures. If thrombosis is present, there is a peripheral hypodense rim surrounding the blood that is flowing [14]. CTA is the preferred modality since it provides information about the feeding artery to the pseudoaneurysm, status of surrounding vessels and structures, and intracranial circulation. MRI, which is not necessary for the diagnosis, can enable visualisation of the pseudoaneurysm and thrombus if present and moreover permit a 3D rendering of the other arteries. Catheter angiography remains the gold standard for the diagnosis despite the fact that it is invasive. It is essential in determining the extent of a thrombus and the presence of arteriovenous fistulas and permits an assessment of collateral circulation [7, 15]. Usually, It is used when therapeutic procedures are being contemplated [9]. It has been recommended that duplex ultrasound could be used for neurologically asymptomatic patients and invasive angiography be reserved for those with deficits, evidence of vessel disruption, or abnormality on duplex scanning [11]. In our setting (low income country), only Doppler ultrasound and CTA are feasible, with the latter being less affordable because of its cost (at least 240 US $).

As stated by Singhal et al., the treatment of pseudoaneurysms depends on its morphology, the segment involved, the age of the patient, and most importantly the status of collateral circulation to brain [9]. However, the economic environment and the technical platform can seriously influence therapeutic choices. The primary goals of treatment are to isolate the pseudoaneurysm from circulation in order to prevent its rupture, to permit distal thromboembolism, and to relieve compression on adjacent vital structures [9].

Three therapeutic options could be considered in the treatment of carotid pseudoaneurysms: conservative management, endovascular treatment, or surgical repair. Conservative management with close long-term follow-up and anticoagulation should be reserved to very small aneurysms [15]. Endovascular treatment includes stent insertion and coiling and parent vessel occlusion. It can be efficiently used particularly when the cerebral circulation is satisfactory through the Willis circle [9, 12]. However, although several authors have already used endovascular treatment successfully [4, 8, 9, 19], it is not considered advisable on children because of insufficient data and some doubts about its long-term outcomes [5, 11]. In this case, surgical approach was chosen for two reasons. Firstly, there was a huge hematoma impairing airway that needed to be evacuated rapidly. The second reason was the level of the lesion, since common carotid artery is accessible to surgical approach without need to manipulate mandibular of other bones and nerves.

Surgical treatment includes five options summarized by Garg et al. [6]: clipping, resection and primary end-to-end anastomosis, resection with graft interposition, extracranial to intracranial bypass, and carotid artery ligation. Carotid artery ligation in children my cause stroke and possibly death if the collateral circulation is insufficient and should be reserved as last resort or in cases of aneurysm rupture. Restoring vascularization after surgical resection by direct anastomosis or bypass is the most recommended approach [5, 6, 13]. For lesions that are not accessible to surgical management, anticoagulation with or without stenting and close follow-up are appropriate [11]. Usually, anticoagulation is considered as a temporary treatment to reduce the embolic risk prior to surgery. The technique often used is resection of the pseudoaneurysm followed by end-to-end anastomosis or insertion of a venous graft [6]. In our case the end-to-end anastomosis was performed.

Several complications can follow the operation: transient ischemic attacks, stroke, cervical or cranial nerve injuries, graft occlusion, or death [5, 6]. Despite those complications, surgical management is the most frequently used option for the treatment of carotid pseudoaneurysm in children and is considered to have the better long-term prognosis. In 2007 Pourhassan et al. described 4 cases of surgically managed pseudoaneurysms in children with favourable outcome after 25-year follow-up [5].

## 4. Conclusion

Carotid artery pseudoaneurysm is an uncommon pathology particularly in the paediatric population. It is mostly due to trauma but can also occur during unexpected circumstances such as food intake. For the treatment, endovascular and surgical options could be considered, each possibility having its precise indications. When possible, surgical management by resection and end-to-end anastomosis would be preferred in order to avoid the risk of compression on neighbouring organs and rupture. This approach seems to provide the most favourable long-term outcome.

## Figures and Tables

**Table 1:** Noninfectious carotid pseudoaneurysms reported in the paediatric population.

Authors and year	Sex and age	Carotid location	Mechanism	Treatment
Mahmoud et al., 2012 [8]	M, 4 years	Left common	Stabbing	Balloon trapping

Binning et al., 2010 [20]	F, 11 weeks	Right internal	Motor vehicle collision	Direct suture of the arterial defect and muslin sling wrapping

Singhal et al., 2009 [9]	M, 2 years M, 8 years	Right internal	Fine needle aspiration of cervical lymph node	Proximal and distal coiling Ligation

Beena et al., 2007 [7]	M, 3 years	Right internal	Needle aspiration of a parotid abscess	Not reported

DeFatta et al., 2005 [17]	F, 8 years	Left internal	Unclear : infection or transoral parapharyngeal abscess drainage	Ligation after coiling failure

Cuff and Thomas, 2005 [11]	M, 12 years	Left common	Elbow-strike during a basketball match	Resection and saphenous graft interposition

Hoff et al., 2005 [21]	M, 7 year	External	Tonsillectomy	Ligation

Chambers et al., 2002 [19]	F, 11 months	Right internal	Fall with a spoon inside the mouth	Coiling

Windfuhr, 2001 [15]	F, 5 years	Left internal	Fall with blunt trauma of head and neck	Ligation

Henriksen et al., 2000 [4]	F, 7 years	Right lateral aberrant internal	Myringotomy	Balloon embolization

Karas et al., 1997 [22]	M, 6 years	Right external	Tonsillectomy	Surgical trapping

Tovi et al., 1987 [13]	F, 14 years	Right internal	Tonsillectomy	Ligation of the sac with proximal and distal coiling

M: male. F: female.
